# Low-grade inflammatory parameters may be associated with recent suicide attempts – a naturalistic study among psychiatric inpatients with depressive disorders

**DOI:** 10.3389/fpsyt.2026.1707768

**Published:** 2026-02-09

**Authors:** Csenge Lovig, Peter Osvath, Eszter Saghy, Csilla Molnar, Marton Aron Kovacs, Borbala Petho, Diana Simon, Sandor Fekete, Tamas Tenyi, Viktor Voros

**Affiliations:** 1 Department of Psychiatry and Psychotherapy, Clinical Center, Medical School, University of Pecs, Pecs, Hungary; 2 Center for Health Technology Assessment and Pharmacoeconomic Research, Faculty of Pharmacy, University of Pecs, Pecs, Hungary; 3 Department of Immunology and Biotechnology, Clinical Center, Medical School, University of Pecs, Pecs, Hungary

**Keywords:** C-reactive protein (CRP), depressive disorders, immune biomarkers, low-grade inflammation, monocyte-to-lymphocyte ratio (MLR), neutrophil-to-lymphocyte ratio (NLR), suicidal behavior, suicide attempt

## Abstract

**Introduction:**

Psycho-neuro-immunological research examines the relationship between mental disorders and immune abnormalities. Chronic, low-grade inflammation was found to be associated with depression and suicidal behavior; however findings are inconsistent due to methodological differences. This study aims to identify peripheral immunological parameters that may characterize patients with recent suicide attempt.

**Method:**

100 psychiatric in-patients treated with depressive disorders with or without recent suicide attempt were investigated. Demographic data, severity of depression, suicide risk and peripheral immunological parameters were recorded. Descriptive statistics and logistic regression analyses were carried out to assess differences between the recent suicide attempter and the non-suicidal groups.

**Results:**

There were no significant differences between recent and non-recent suicide attempters in age, gender, or depression severity. However, recent suicide attempters exhibited higher levels of leukocytes, neutrophils, eosinophils, monocytes, Neutrophil-to-Lymphocyte Ratio (NLR) and Monocyte-to-Lymphocyte Ratio (MLR). NLR and MLR remained elevated in the recent suicide attempter group after adjusting for confounders. Significant positive correlations were observed between NLR, MLR, Platelet-to-Lymphocyte Ratio (PLR), and platelet values, but not with CRP, while depression scores correlated negatively with PLR, NLR, and MLR.

**Conclusion:**

Although elevated low-grade inflammatory parameters, particularly NLR and MLR, were observed in depressed patients with a recent suicide attempt, this study does not provide conclusive evidence for a direct association between suicide attempts and the investigated immune markers. Further research is needed to clarify this relationship between low-grade immune parameters and acute suicide attempts, to elucidate potential causal mechanisms, and to determine their relevance and clinical applicability as biomarkers of suicidal behavior.

## 1 Highlights

- This study examined peripheral low-grade inflammatory markers (NLR, MLR, PLR, CRP) in 100 depressed inpatients with and without a recent suicide attempt.
- Higher NLR and MLR values were observed in patients with a recent suicide attempt compared to those without.
- Depression severity showed negative associations with PLR, while lifetime suicide risk was negatively correlated with NLR and MLR.
- CRP levels did not differ significantly between groups and showed minimal correlation with other immune markers, suggesting a limited association with acute suicide attempt.
- Overall, the findings indicate a possible—but not conclusive—association between elevated low-grade inflammatory markers (particularly NLR and MLR) and recent suicide attempts, underscoring the need for further research prior to clinical application.

## 2 Introduction

A growing body of evidence highlights the significance of certain immune and inflammatory parameters linked to suicidal behavior (1). Research has investigated abnormalities in central and peripheral immunological regulatory systems in relation to suicidal behavior to identify peripheral biomarkers that may predict suicidal risk (2, 3). Numerous pathophysiological consequences of the chronic inflammatory process have been confirmed, including dysregulation of the Hypothalamic–Pituitary–Adrenal (HPA) axis and the role of pro-inflammatory cytokines (4). Additionally, it has been established that the neuronal deficit resulting from immunological and inflammatory neurotoxicity plays a role not only in the development of depressive and psychotic symptoms, but also in suicidal behavior (5).

Low-grade inflammatory parameters such as Neutrophil-to-Lymphocyte Ratio (NLR), Monocyte-to-Lymphocyte Ratio (MLR) and Platelet-to-Lymphocyte Ratio (PLR), as peripheral biomarkers can be measured by routine laboratory tests. An increase in NLR and MLR was found in patients with Bipolar Disorder (BPD) (6) and Major Depressive Disorder (MDD) (7, 8). In a study conducted with a population of more than thirty-thousand adults, U-shaped non-linear relationships and threshold effects were observed between PLR and depression and symptom severity (9). Elevated MLR and PLR levels were also confirmed in a population of Non-Suicidal Self-Injurers (NSSI), however NLR elevation was not significant (10).

There has been a growing interest in psycho-neuro-immunological research examining the relationship between peripheral immune abnormalities and suicidal behavior. Their significance lies in their possibility of identifying immune-related and inflammatory biomarkers associated with suicidal behavior (11, 12). Several studies have confirmed the correlation between NLR and suicidal vulnerability in patients with BPD and MDD (13). In patients with MDD, NLR has been suggested to be a trait marker for suicidal vulnerability via a relationship between NLR and a recent suicide attempt in depressed inpatients (14). Velasco (12) also reported higher NLR and PLR levels among depressed suicidal patients compared to non-suicidal depressed patients. However, these parameters did not differ between current and previous suicide attempters. Furthermore, higher NLR levels were found to distinguish violent and non-violent suicide attempters (11, 15). In the latter study, however, no significant differences were found in inflammatory parameters except for increased NLR. Aguglia (16) found that platelet size and PLR were significantly correlated with the lethality of the suicide attempt. Akkus (17) also reported elevated NLR and PLR measures in a sample of suicide attempters with drug-overdose, which significantly decreased after hospitalization. These studies suggest that the measurement of NLR may serve to be a cost-effective method to more accurately assess suicidal risk in MDD patients. However, conflicting evidence exists, as Meydaneri (18) found no significant difference in NLR, PLR and other immune biomarkers between suicidal patients and non-suicidal patients with depressive disorders. Furthermore, Capuzzi (19) reported opposite correlation, observing that NLR and lipid parameters, such as Cholesterol, Low-Density Lipoprotein (LDL) and Very Low-Density Lipoprotein (VLDL) were significantly lower in violent suicide attempters compared to non-violent attempters. The authors contributed these contradictory findings to differences in methodology and sample selection.

In addition to the above peripheral immunological parameters, the relationship between C-Reactive Protein (CRP) - which is also a marker of inflammation - and suicidal behavior was also investigated. Courtet (20) observed a significant increase in the CRP value in six-hundred patients with depression, who attempted suicide either currently or during their lifetime. It was suggested that the CRP increase in patients with depression is not only the result of the stress caused by an acute suicidal act but can also be a trait marker of suicidal vulnerability. The correlation between CRP elevation and suicidal behavior has been confirmed by several other studies (14, 21). Aguglia (22) also found significantly elevated CRP levels, but only in violent suicide attempters. These results suggest that there may be more pronounced inflammation in this group, which can be related to impulsive-aggressive behavior (16, 22). A meta-analysis based on 21 studies (7,682 people, a significant majority with mental disorders) found significant relationship between elevated CRP and suicidality (5). This correlation was confirmed both for suicide intentions and attempts. A meta-analysis of 36 studies (2,679 suicide attempters and 6,839 controls) also found a correlation between CRP elevation and current suicide attempt (1). According to their results, the observed immunological and inflammatory abnormalities - as state-dependent markers - are directly related to suicidal behavior, regardless of the underlying mental disorder. The above research supports the possibility that immunological laboratory parameters may be used to screen patients with suicidal risk in the depressed population (20), and that CRP may possibly be a transdiagnostic marker of suicidal risk (5).

Although previous findings are somewhat contradictory due to methodological differences, there is growing evidence that certain immunological parameters related to low-grade inflammation, particularly elevated NLR and CRP are associated with MDD and suicidal behavior. Building on this, we designed a study to assess immunological parameters that may characterize patients who had recently attempted suicide with drug-overdose and were admitted to a psychiatric ward. This naturalistic study aimed to determine whether routine laboratory immune parameters show detectable alterations in patients presenting for acute psychiatric care after a suicide attempt, and therefore focused on such laboratory and clinical variables that can be routinely assessed in clinical practice. In addition, we investigated the potential relationship between low-grade immunological factors and the severity of depression.

The main questions of this research are the following:

1. Are there differences in the immune profiles between depressed patients who have recently attempted suicide and those who did not have a current suicide attempt?
2. Which low-grade immunological parameters show the strongest association with a recent suicide attempt?
3. How do factors such as gender, age, life-time suicide risk, and the severity of depressive symptoms affect the immunological parameters of depressed inpatients?

## 3 Methods

### 3.1 Study population

The study population included psychiatric in-patients (N = 100) consecutively treated with depressive episode with or without a current suicide attempt in a University Clinic between December 1, 2020, and December 31, 2021. The sample consisted of 36 individuals who had recently attempted suicide (recent suicide attempters) and 64 patients who had never attempted or had previously attempted suicide (patients without recent suicide attempt). Recent suicide attempters were defined as those patients, who were hospitalized due to an acute suicide attempt. Only those patients were included in the recent suicide attempters’ group, who attempted suicide by drug-overdose, in order to ensure a more homogeneous study population and to avoid the inclusion of individuals whose method of suicide attempt (e.g., cutting, stabbing, jumping, hanging, etc.) could directly affect immunological and inflammatory parameters.

Patients with current active and intense suicidal ideations or with a suicide attempt within one month were also excluded, except for those who had made an attempt within the preceding three days of admission, as they were included in the recent suicide attempter group.

Furthermore, patients with acute infections (e.g., tonsillitis, pneumonia) and with chronic immunological–rheumatological (e.g., systemic lupus erythematosus, Sjögren’s syndrome), musculo-skeletal (e.g., rheumatoid arthritis, Bechterew disease), or central nervous system (e.g., multiple sclerosis, myasthenia gravis) diseases or who were treated with anti-inflammatory medications (e.g., corticosteroids and Non-Steroid Anti-Inflammatory Drugs (NSAIDs)) were excluded. Patients who had previously received or were currently receiving specific immunological treatments were also excluded from the study.

### 3.2 Study participation

Participation in the study was voluntary, and no study-related procedures were completed before signing the Informed Consent Form (ICF). Following the signature of the ICF, a self-reported questionnaire was completed besides a semi-structured interview. All study-related procedures were conducted within three days following the suicide attempt and also within three days of hospital admission in the group without a current suicide attempt. This narrow inclusion time-frame was applied to ensure group homogeneity and to include only those patients who had recently attempted suicide. The absence of current suicide attempts in the group of patients without a recent suicide attempt was verified to ensure that none of these patients had engaged in a suicide attempt within the preceding month.

Patients who met the eligibility criteria were invited to participate in the study. A total of 111 patients were offered participation based on the inclusion and exclusion criteria, of whom 100 agreed to take part and signed the ICF. Three patients who had recently attempted suicide and eight patients without recent suicide attempt refused to sign the ICF and to participate in the study, primarily due to constraints in time, interest, or lack of motivation. After reaching 100 eligible patients who provided informed consent, patient recruitment was discontinued.

Participation or non-participation in the study did not affect the clinical treatment of the patients in any ways. The study was approved by the Central Ethics Committee (Medical Research Council, Scientific and Research Ethics Committee) and the local Institutional Review Board (IRB) under the approval number No.8579-PTE/2020.

### 3.3 Data collection

Basic demographic data, severity of depression, suicide risk and immunological parameters (white blood cell count: lymphocytes, monocytes, neutrophil, eosinophil, basophil granulocytes; platelets; hs-CRP) were recorded. The assessment of the immunological parameters was carried out as part of the routine laboratory examinations on the day following admission. Based on the results of these laboratory tests, the Neutrophil-to-Lymphocyte Ratio (NLR), the Monocyte-to-Lymphocyte Ratio (MLR), and Platelet-to-Lymphocyte Ratio (PLR) were calculated.

The Patient Health Questionnaire - 9 item (PHQ-9) and the Suicide Risk Assessment Scale (SRAS) were used to gather information about the severity of depression and life-time suicide risk, respectively (23–25). The PHQ-9 is the depression module of the PHQ, which is a self-administered version of the PRIME-MD diagnostic instrument for common mental disorders (23). This multipurpose instrument scores each of the 9 DSM criteria for depression as “0” (not at all) to “3” (nearly every day), and is widely used for screening, diagnosing, monitoring and measuring the severity of depression (23). PHQ-9 total scores represent no or minimal (0 to 4), mild (5 to 9), moderate (10 to 14), moderately severe (15 to 19), and severe (20 to 27) depression (23). The SRAS is a suicide risk assessment tool developed by Rihmer (24, 25) based on the hierarchical classification of suicide risk factors, including, primary (psychiatric and medical), secondary (psycho-social) and tertiary (demographic) risk factors and also protective factors. This hierarchical scoring system (3 points for primary, 2 points for secondary, and 1 point for tertiary risk factors) summarizes the most important suicide risk (mental disorders, previous suicide attempt, suicide in the family, etc.) and protective (living with children, pregnancy, being religious, etc.) factors and allows for an assessment of the relative weight of individual factors. Higher scores indicate higher suicide risk with total scores represent no or mild (0 to 8), moderate (9 to 14) or severe (15 to 32) suicide risk (25).

### 3.4 Statistical analysis

For the two participant groups (recent suicide attempters and non-recent suicide attempters), we conducted a descriptive analysis covering age, gender, scores derived from clinical questionnaires (PHQ-9, SRAS), and laboratory findings. To identify statistical differences between the groups, the Wilcoxon rank-sum test was used for continuous variables, while Pearson’s chi-squared test and Fisher’s exact test were applied to ordinal and categorical variables. P-values were adjusted for multiple comparisons using the Benjamini–Hochberg false discovery rate (FDR) procedure.

Given the relatively small sample size and the non-normal distribution of several continuous variables, we consistently used non-parametric tests throughout the analysis to avoid assumptions of normality. No values were excluded or trimmed in order to retain statistical power and reflect real-world variability.

Logistic regression analyses were carried out on the binary variable representing suicide attempts, where ‘1’ denoted individuals in the recent suicide attempter group and ‘0’ represented those who had not attempted suicide recently (previously attempted suicide or never attempted suicide). A *p*-value below 0.05 was considered to indicate statistical significance. All statistical analyses were conducted using R version 4.2.3 within the RStudio integrated development environment (Version 2023.03.0 + 386).

## 4 Results

The most frequent clinical diagnoses were Major Depressive Disorder (MDD), single (F32) (N = 36) or recurrent episode (F33) (N = 11), Mixed Anxiety and Depressive Disorder (MADD) (F4120) (N = 34), and Bipolar Disorder (BPD) depressive episode (F313-F315) (N = 19) according to the ICD-10 diagnostic criteria, based on a semi-structured clinical interview (WHO, 2004) (32). The prevalence of comorbidity among patients was widespread, affecting 95 individuals in total. The most frequent comorbidities were Adjustment Disorder (AD) (F4320) (N = 44), Personality Disorders (PDs) (F60) (N = 23), and Alcohol Use Disorders (AUDs) (F1010-F1020) (N = 19).

The demographic and clinical characteristics of the study population are summarized in **Table 1**. There was no significant difference between the population of recent suicide attempters and non-recent suicide attempters in terms of age, gender, and severity of depression. Among the immunological parameters, several markers —including leukocyte, neutrophil, eosinophil, and monocyte counts, as well as NLR and MLR— showed higher unadjusted values in the recent suicide attempter group compared to the non-recent suicide attempter group. However, these differences did not remain statistically significant after correction for multiple comparisons using the Benjamini–Hochberg false discovery rate procedure (**Table 2**). No significant group difference was observed in CRP levels.

**Table 1:** Demographic and clinical characteristics of the non-recent and the recent suicide attempter groups.

Variable	Non-recent suicide attempters (N = 64)	Recent suicide attempters (N = 36)	Significance (Raw p)
*Sex*			0.246
*Male (%)*	18 (28%)	15 (42%)	
*Female (%)*	46 (72%)	21 (58%)	
*Age*	47.5 [29.5]	50.5 [25.25]	0.391
*Depression (PHQ-9 total)*	15.5 [9.75]	12 [9]	0.35
*Suicide risk (SRAS)*	13 (21%)	6 (17%)	0.799

NS, Non-significant; PHQ-9, Patient Health Questionnaire - 9 item; SRAS, Suicide Risk Assessment Scale.

The Sex variable was assessed using the Pearson’s Chi-squared test, the Age, Suicide risk, and Depression variables were assessed using the Wilcoxon rank-sum test. *The table presents median [IQR] for continuous and n (%) for categorical variables.*

**Table 2:** The immunological parameters in the non-recent and the recent suicide attempter groups.

Variable	Non-recent suicide attempters (N = 64)	Recent suicide attempters (N = 36)	Raw p	FDR-adjusted p
Leukocyte	6.79 [2.82]	7.41 [3.44]	0.047	0.094
Neutrophil	4.03 [2.38]	4.80 [3.41]	0.023	0.06
Lymphocyte	2.03 [0.96]	1.94 [0.69]	0.181	0.295
Monocyte	0.37 [0.16]	0.54 [0.35]	0.013	0.06
Eosinophil	0.13 [0.12]	0.08 [0.11]	0.02	0.06
Basophil	0.04 [0.02]	0.03 [0.04]	0.503	0.544
Platelet	263 [83]	227.5 [84.25]	0.258	0.373
NLR	1.93 [1.16]	2.83 [2.41]	0.021	0.06
MLR	0.18 [0.08]	0.28 [0.23]	0.01	0.06
PLR	128.25 [57.67]	114.86 [71.90]	0.884	0.884
CRP	0.80 [3.60]	2.25 [6.85]	0.051	0.094

CRP, C-Reactive Protein; IQR, Inter-Quartile Range; MLR, Monocyte-to-Lymphocyte Ratio; NLR, Neutrophil-to-Lymphocyte Ratio; NS, Non-significant; PLR, Platelet-to-Lymphocyte Ratio.

Significance between the groups were tested using the Wilcoxon rank-sum test. *The table presents median [IQR] values.*

**Table 3** presents the results of multiple logistic regression models predicting recent suicide attempts (1 = recent attempter, 0 = non-recent or no attempt). Each model included one inflammatory marker (platelet, NLR, MLR, PLR, or CRP) along with covariates for age, gender, continuous depression severity (PHQ-9), and binary cumulative suicide risk. Among the models, only MLR was a statistically significant predictor of recent suicide attempts (OR = 32.68, 95% CI [1.64, 1185.77], p = .034). The remaining markers did not significantly predict recent suicide attempts after adjusting for covariates.

**Table 3:** Multiple logistic regression results for the predictors of recent suicide attempt.

Model	Predictors	B	SE	Wald χ²	p	OR	95% CI for OR	Nagelkerke R²	Hosmer–Lemeshow χ²(df=8), p
1. Platelet	Platelet	-0.001	0.004	0.151	0.698	0.999	[0.991, 1.006]	0.421	χ² = 13.719, p = .089
	Female	-0.149	0.555	0.072	0.788	0.862	[0.290, 2.604]		
	Age	0.009	0.014	0.404	0.525	1.009	[0.981, 1.039]		
	Depression (PHQ-9)	-0.024	0.04	0.377	0.539	0.976	[0.901, 1.056]		
	Suicide risk (SRAS)	-0.158	0.665	0.056	0.813	0.854	[0.209, 3.011]		
2. NLR	NLR	0.219	0.12	3.308	0.069	1.245	[1.008, 1.646]	0.462	χ² = 9.985, p = .266
	Female	-0.091	0.574	0.025	0.873	0.913	[0.298, 2.896]		
	Age	0.004	0.015	0.063	0.801	1.004	[0.974, 1.034]		
	Depression (PHQ-9)	-0.009	0.042	0.048	0.827	0.991	[0.913, 1.077]		
	Suicide risk (SRAS)	-0.013	0.672	0	0.985	0.987	[0.239, 3.527]		
3. MLR	MLR	3.246 *	1.603	4.102	0.043	25.696	[1.393, 856.971]	0.468	χ² = 4.351, p = .824
	Female	0.12	0.595	0.04	0.841	1.127	[0.358, 3.786]		
	Age	-0.002	0.016	0.02	0.887	0.998	[0.967, 1.029]		
	Depression (PHQ-9)	-0.012	0.041	0.079	0.779	0.988	[0.911, 1.074]		
	Suicide risk (SRAS)	-0.059	0.673	0.008	0.931	0.943	[0.228, 3.370]		
4. PLR	PLR	0.005	0.003	1.955	0.162	1.005	[0.998, 1.012]	0.441	χ² = 20.885, p = .007
	Female	-0.175	0.562	0.097	0.756	0.84	[0.279, 2.577]		
	Age	0.007	0.015	0.224	0.636	1.007	[0.978, 1.037]		
	Depression (PHQ-9)	-0.014	0.041	0.111	0.739	0.986	[0.909, 1.071]		
	Suicide risk (SRAS)	-0.103	0.669	0.024	0.878	0.902	[0.219, 3.201]		
5. CRP	CRP	0.03	0.036	0.704	0.401	1.031	[0.959, 1.112]	0.427	χ² = 10.509, p = .231
	Female	-0.298	0.57	0.273	0.601	0.742	[0.241, 2.297]		
	Age	0.008	0.015	0.283	0.595	1.008	[0.979, 1.037]		
	Depression (PHQ-9)	-0.019	0.041	0.212	0.645	0.981	[0.905, 1.064]		
	Suicide risk (SRAS)	-0.123	0.666	0.034	0.853	0.884	[0.216, 3.122]		

CRP, C-Reactive Protein; MLR, Monocyte-to-Lymphocyte Ratio; NLR, Neutrophil-to-Lymphocyte Ratio; PHQ-9, Patient Health Questionnaire - 9 item; PLR, Platelet-to-Lymphocyte Ratio; SRAS, Suicide Risk Assessment Scale.

Coefficients marked with an asterisk (*) are statistically significant at the p <0.05 level.

To test the robustness of the findings, a sensitivity analysis was conducted in which participants who were currently depressed and had a history of past (non-recent) suicide attempts were excluded. Re-estimating the logistic regression model on this restricted sample produced a similar effect size for MLR, although the association was not statistically significant (B = 3.21, SE = 1.64, p = .050). The corresponding odds ratio suggested a large effect (OR = 24.74, 95% CI [1.24, 873.93]), but with wide confidence intervals reflecting reduced precision. None of the covariates reached statistical significance. Model fit remained acceptable (Nagelkerke R² = .453), and the Hosmer–Lemeshow test indicated good calibration (χ²(8) = 6.50, p = .592).

**Figure 1:**
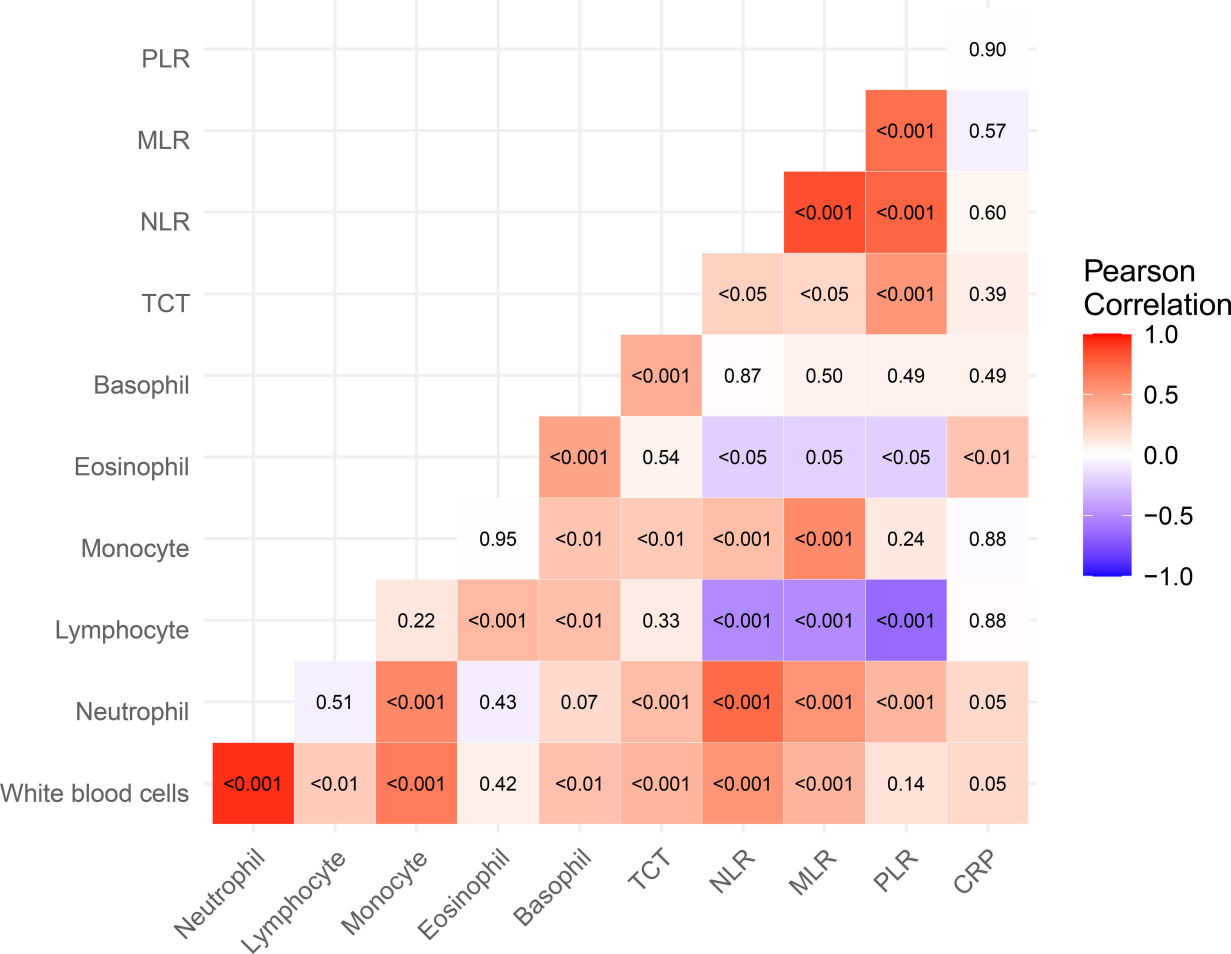
Heat-map representing the Pearson’s correlation matrix of various immunological parameters. CRP, C-Reactive Protein; MLR, Monocyte-to-Lymphocyte Ratio; NLR, Neutrophil-to-Lymphocyte Ratio; PLR, Platelet-to-Lymphocyte Ratio; TCT, Thrombocyte (Platelet). Each square in the heat-map corresponds to the correlation between two specific immunological parameters. The heat-map uses a color gradient to represent the strength and direction of these correlations: red indicates a positive correlation, blue a negative correlation. Each square in the heat-map contains a p-value, reflecting the statistical significance of the corresponding correlation. P-values less than 0.05, 0.01, and 0.001 are denoted by “<0.05”, “<0.01”, and “<0.001”, respectively, signifying a progressively stronger rejection of the null hypothesis of no correlation.

**Figure 1** shows the heat-map representing the Pearson’s correlation matrix of various immunological parameters, with adjustments made to control for the effects of age and gender. There are strong significant (*p* < 0.001) positive correlations between NLR and MLR, NLR and PLR, and PLR and MLR values. CRP was not significantly correlated with any other immunological parameters, except for eosinophil granulocytes (*p* < 0.01).

**Table 4** presents the results of individual proportional odds logistic regression models, examining the impact of various immunological parameters on depression (PHQ-9) and life-time suicide risk (SRAS), while controlling for age and gender. Each immunological parameter is analyzed separately in its own regression model to mitigate potential multicollinearity issues arising from the substantial correlations among these variables. Positive correlation between depression and suicide risk were not found, but there were significant negative associations between PLR and depression, and between NLR, MLR and life-time suicide risk.

**Table 4:** Individual proportional odds logistic regression models of the effect of immunological parameters on depression and life-time suicide risk.

	PHQ (depression)		SRAS (suicide risk)	
	Coefficient	P-value	Coefficient	P-value
*WBC*	0.08	0.935	-1.81	0.071
*Neutrophil*	-0.59	0.557	-2.55	0.011
*Lymphocyte*	1.56	0.119	1.61	0.108
*Monocyte*	0.38	0.705	-1.48	0.138
*Eosinophil*	0.47	0.641	0.92	0.359
*Basophil*	0.72	0.473	0.56	0.575
*Platelet*	0.03	0.979	-0.33	0.741
*NLR*	-1.89	0.059	**-2.59**	**0.010**
*MLR*	-1.54	0.124	**-2.18**	**0.029**
*PLR*	**-1.96**	**0.050**	-1.83	0.067
*CRP*	-1.70	0.090	0.28	0.780

CRP, C-Reactive Protein; MLR, Monocyte-to-Lymphocyte Ratio; NLR, Neutrophil-to-Lymphocyte Ratio; PLR, Platelet-to-Lymphocyte Ratio; WBC, White Blood Cell.

The bold values indicate statistically significant negative associations: between PLR and depression severity, as well as between NLR, MLR and lifetime suicide risk.

## 5 Discussion

### 5.1 Findings

In line with previous research, our findings show that several peripheral low-grade inflammatory parameters - specifically leukocyte, neutrophil granulocyte, and monocyte count, as well as the Neutrophil-to-Lymphocyte Ratio (NLR) and Monocyte-to-Lymphocyte Ratio (MLR) - were higher in depressed patients with current suicide attempt compared to those without a recent attempt (1, 7, 12, 14, 15), although these differences were not statistically significant. Regression analysis further demonstrated that MLR remained significantly elevated in the recent suicide attempter group after adjusting for covariates and controlling for age, gender, life-time suicide risk, and severity of depression; however, wide confidence intervals indicate unstable effect estimates and potential overfitting. However, low-grade inflammatory parameters showed a close correlation with each other as well, consistent with previous studies (12, 17). Our findings suggest that the combined elevation of these low-grade immune parameters may be associated with acute suicide attempts, reflecting a low-intensity, non-specific, and complex immunological mechanism. However, these findings should be considered exploratory rather than definitive evidence for suicide-related low-grade inflammatory immune biomarkers.

CRP levels were also higher in recent suicide attempters; however, the difference was not statistically significant. Furthermore, CRP level was significantly correlated only with eosinophil granulocyte count. These results are not clearly supportive for previous findings on eosinophil count and CRP as markers of suicidal behavior (16, 20, 21).

Firstly, in their meta-analysis, Miola (5) reported a significantly stronger correlation between elevated CRP levels and suicidal ideation than with suicide attempts in populations with severe mental disorders. However, other studies have found no significant association between CRP and suicidal ideation (20, 26). One possible explanation raised in the literature is that CRP may represent a non-specific inflammatory marker that is associated with a range of other psychiatric conditions as well (5, 27). However, our study investigated recent suicide attempters with drug-overdose and focused mainly on state-markers; but other previous research reported CRP as a potential trait-marker of suicide attempt (20) or found significant CRP level elevations in patients with high-lethality suicide attempts (16). Previous studies have also indicated that more pronounced elevations in CRP are primarily observed in individuals with more severe depressive symptomatology (28). The relatively modest increase in CRP levels observed in our sample may be explained by the fact that only 33% of the study population met criteria for severe depression (PHQ-9 ≥ 20). A possible explanation for this finding is that elevated CRP levels may be more closely associated with severe forms of depressive mood disorders -or with a specific inflammatory subtype thereof- rather than with suicidal behavior per se (28). In contrast, our results suggest that cellular immune markers such as the NLR and MLR may be more specifically correlated with recent suicide attempts (29).

Secondly, based on the systematic review and meta-analysis by Daray et al., the eosinophil count is not associated with increased or decreased risk of suicidal ideation or suicidal behavior, either in the general population or among individuals diagnosed with depression (29). This contrasts with other immune markers like neutrophil count, WBC count, and NLR, which were significantly elevated in individuals with suicidal ideation or behavior. However, in our study, CRP was not significantly correlated with any other immunological parameters, except for eosinophil granulocytes, suggesting a potentially distinct regulatory pathway that may merit further investigation (29).

A particularly noteworthy finding is that these immunological parameters showed negative associations with the severity of depression (PLR) and life-time suicide risk (NLR, MLR). Several possible explanations may account for this result. One possibility is that these immunological alterations exist on a pathophysiological continuum, where milder changes are associated with depressive symptoms, and the most pronounced deviations are linked to suicidal behavior (29). However, our results do not support this interpretation, as NLR and MLR were found to correlate negatively with the severity of depression. An alternative explanation is that suicidal behavior may represent a distinct entity characterized by non-specific immunological alterations that only partially overlap with those observed in depression. A third possibility is the existence of a specific subgroup among depressed individuals - so-called ‘immunodepression’ - characterized by increased immune cell counts and a higher propensity for suicidal behavior (29).

In summary, our findings align with previous data suggesting that recent suicide attempters – who exhibit a combined increase of different low-grade immune-related biomarkers – may represent a distinct sub-group within patients with depression (1, 12, 29). Regarding the potential complex pathophysiological mechanisms underlying the association between inflammatory and immunological parameters, depression, and suicidal behavior we refer to recent literature (1, 20, 21, 29, 30). These mechanisms include immune system dysregulation and neuroinflammatory processes (e.g. CRP, interleukins and TNF-α alterations) that may contribute to serotonergic depletion, disturbances in NMDA neurotransmission, elevated glucocorticoid levels, dysregulation of the HPA axis, as well as decreased neurogenesis and neuroplasticity (28–30). In addition, dysfunction of the prefrontal cortex has been implicated, potentially leading to cognitive impairments such as deficits in decision-making, increased impulsivity, emotional dysregulation, and hopelessness, all of which may increase the risk of suicidal behavior (31).

### 5.2 Strengths and limitations

The major limitations of the study include its cross-sectional design with a small sample size, which merely captures a snapshot of the depressed patients’ current psychological and neuro-biological state. This restricts our ability to draw conclusions on the causal relationship between suicidal behavior and low-grade inflammation. The relatively small number of cases in both the recent suicidal and non-suicidal groups is another limitation, though clinically meaningful immunological differences were detected despite the small sample size. Furthermore, due to the low number of patients with a lifetime history of suicide attempts (N = 19) in the non-recent suicide attempter group, it was not feasible to analyze this group separately as a distinct subgroup. Finally, the study did not include a control group of healthy individuals. In future studies with larger sample sizes, it would be beneficial to examine and compare these groups (i.e., individuals with an acute suicide attempt, individuals with a past suicide attempt, and individuals with no history of suicide attempts), and to compare them with both a group of patients with depression and the general population.

One of the strengths of the study is its naturalistic methodology, including nearly all consenting depressed patients admitted to the psychiatric department during the study period. Furthermore, a wide range of immunological parameters were measured during the routine laboratory tests, and patients suffering from acute or chronic inflammatory diseases or those taking anti-inflammatory drugs or specific immunological treatments were excluded. By not including violent suicide attempters, only patients with drug overdose, we minimized the potential inflammatory effects of possible injuries (21). However, it cannot be ruled out that drug overdose -depending on the specific type of medication- may exert some systemic inflammatory or immunological effects that are independent of suicidal behavior. Similarly, it should be noted that prior regular psychopharmacological or other pharmacological treatments (except for anti-inflammatory drugs) were not controlled for in the study, although such medications may also potentially influence immunological and inflammatory processes.

In addition to the potential confounder effect of overdose and pharmacological treatment, comorbid mental disorders -which were common among the patients in our sample- such as Personality Disorders, Substance Use Disorders (including Alcohol Use Disorder), and other psychiatric conditions beyond the depressive episode, were not controlled in the analysis, but may also influence inflammatory and immunological markers, potentially confounding the results and limiting their interpretability. Furthermore, Adjustment Disorder was also relatively common in the study population, and theoretically, acute stress response associated with this diagnosis may also have substantial impact on immunological parameters. Besides comorbid mental disorders, common and relatively frequent clinical conditions such as diabetes, hypertension, obesity (increased Body Mass Index (BMI)) or smoking may also be associated with systematic inflammation and therefore influence immunological parameters and suicidal ideations. These conditions were not exclusion criteria for participation in the study, and the corresponding variables were not controlled for in the statistical analyses as there were insufficient clinical data available for these factors to allow their appropriate inclusion in the analysis, which represents a significant limitation. However, since the prevalence of comorbid psychiatric disorders and common clinical conditions above did not differ substantially between the two study groups (those with and without a recent suicide attempt), our findings suggest that the observed low-grade immunological alterations may be more likely to be associated with the recent suicidal behavior rather than the presence of comorbid conditions. Furthermore, it is to be highlighted that the results of the study - due to its naturalistic design and the diagnostic heterogeneity of the depressive clinical study population - may be particularly important from the perspective of the transdiagnostic nature of suicidal behavior.

All continuous variables were inspected for univariate outliers using boxplots and standardized z-scores. While some extreme values were identified, none were removed in order to preserve statistical power given the limited sample size. All observed values fell within plausible clinical ranges.

Blood draw for immunological parameters was completed immediately after admission and the questionnaires and all assessments were also taken within three days, ensuring the accurate record of the current characteristics and minimizing the potential influence of the pharmacotherapeutic and psychotherapeutic interventions, but avoiding the potential cognitive effects of intoxication caused by the medication overdose used for the suicide attempt.

Finally, it is important to emphasize that common methodological challenge in suicide research, that data in the recent suicide attempter group were collected after the suicide attempt, and therefore they reflect the pre-attempt psychological, physical and immunological state only partially. Consequently, our findings may have only limited relevance for suicide risk prediction. However, the reliability of our results is enhanced by the fact that age, gender, depression, and life-time suicide risk were controlled in the statistical model, and that the study focused on psychiatric in-patients with depression, which represent a particularly high-risk group in terms of suicidal behavior, offering valuable real-world clinical insights.

### 5.3 Conclusion

Despite the relatively small sample size and other limitations, our findings suggest a statistically non-significant association between certain low-grade immunological parameters (particularly NLR, MLR) and recent suicide attempt by drug-overdose in inpatients with depressive disorders. These inflammatory parameters showed negative correlations with depression severity and with life-time suicide risk. These observations may point to a potential relationship between immunological mechanisms and current suicide attempt, independent of the underlying mental disorders. The combined elevation of blood-based immunological parameters - regardless of the severity of depression - may indicate psycho-neuro-immunological abnormalities associated with a recent suicide attempt, rather than reflects life-time suicide risk. However, the present study was inconclusive with regard to whether the observed immunological alterations in patients with a recent suicide attempt represent state or trait markers. Although, monitoring these routine inflammatory parameters could serve as an easy-to-use additional tool in the comprehensive assessment of suicide risk in patients with depressive disorders (12, 17), the current evidence remains insufficient to support their routine clinical application. At present, there are no widely accepted, evidence-based guidelines for the routine screening (even in high-risk groups) or treatment (e.g. with anti-inflammatory drugs) of suicidal behavior that are based on the immunological theory of suicide. Given the limited understanding of the causal relationship between suicidal vulnerability and low-grade inflammation, further prospective studies with larger sample sizes and multivariable analytical approaches are warranted to clarify the underlying mechanism of this association and the clinical relevance of these findings.

## Data Availability

The raw data supporting the conclusions of this article will be made available by the authors, without undue reservation.
