# Supplementary material for: Low-grade inflammatory parameters may be associated with recent suicide attempts – a naturalistic study among psychiatric inpatients with depressive disorders
Source: Front Psychiatry. 2026 Feb 9;17:1707768. doi: 10.3389/fpsyt.2026.1707768 (PMC12926131; doi:10.3389/fpsyt.2026.1707768)
Supplement: Supplementary file 1 [file Table1.docx]

**Highlights:**

1. This study examined peripheral low-grade inflammatory markers (NLR, MLR, PLR, CRP) in 100 depressed inpatients with and without a recent suicide attempt.
2. Higher NLR and MLR values were observed in patients with a recent suicide attempt compared to those without.
3. Depression severity showed negative associations with PLR, while lifetime suicide risk was negatively correlated with NLR and MLR.
4. CRP levels did not differ significantly between groups and showed minimal correlation with other immune markers, suggesting a limited association with acute suicide attempt.
5. Overall, the findings indicate a possible—but not conclusive—association between elevated low-grade inflammatory markers (particularly NLR and MLR) and recent suicide attempts, underscoring the need for further research prior to clinical application.
