# Supplementary material for: Low-grade inflammatory parameters may be associated with recent suicide attempts – a naturalistic study among psychiatric inpatients with depressive disorders
Source: Front Psychiatry. 2026 Feb 9;17:1707768. doi: 10.3389/fpsyt.2026.1707768 (PMC12926131; doi:10.3389/fpsyt.2026.1707768)

INTRODUCTION

- Chronic, low-grade inflammation was found to be associated with depression and suicidal behavior
- To identify easy-to-detect peripheral immune parameters associated with recent suicide attempts among patients with depression.

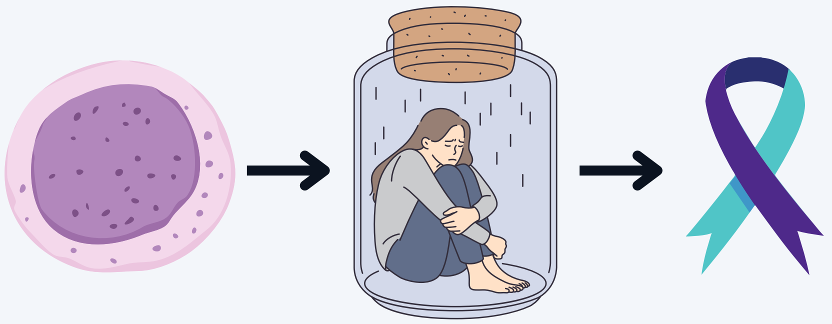

METHODOLOGY

- Participants: 100 psychiatric inpatients with depressive disorders (with/without recent suicide attempt)
- Assessments: PHQ-9, Suicide Risk Assessment Scale, Immune Markers
- Analysis

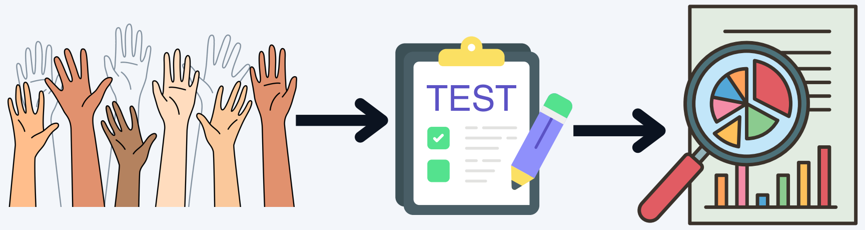

RESULTS

- Recent suicide attempters with depressive disorders showed a distinct inflammatory profile characterized by elevated low-grade immune markers.

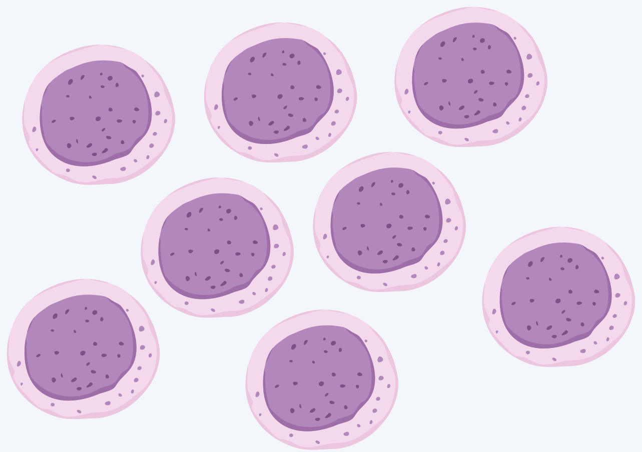

CONCLUSION

- Our findings suggest a possible link between elevated low-grade inflammatory markers (especially NLR and MLR) and recent suicide attempts, independent of depression severity or lifetime suicide risk.
- However, current evidence is insufficient to support their routine clinical use, and further large-scale studies are needed.

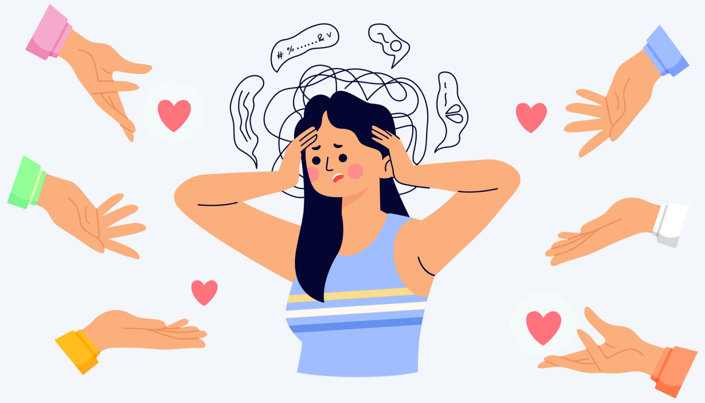

Supplement: Supplementary file 2 [file DataSheet1.pdf]
