# Supplementary figures and images for: Low-grade inflammatory parameters may be associated with recent suicide attempts – a naturalistic study among psychiatric inpatients with depressive disorders
Source: Front Psychiatry. 2026 Feb 9;17:1707768. doi: 10.3389/fpsyt.2026.1707768 (PMC12926131; doi:10.3389/fpsyt.2026.1707768)

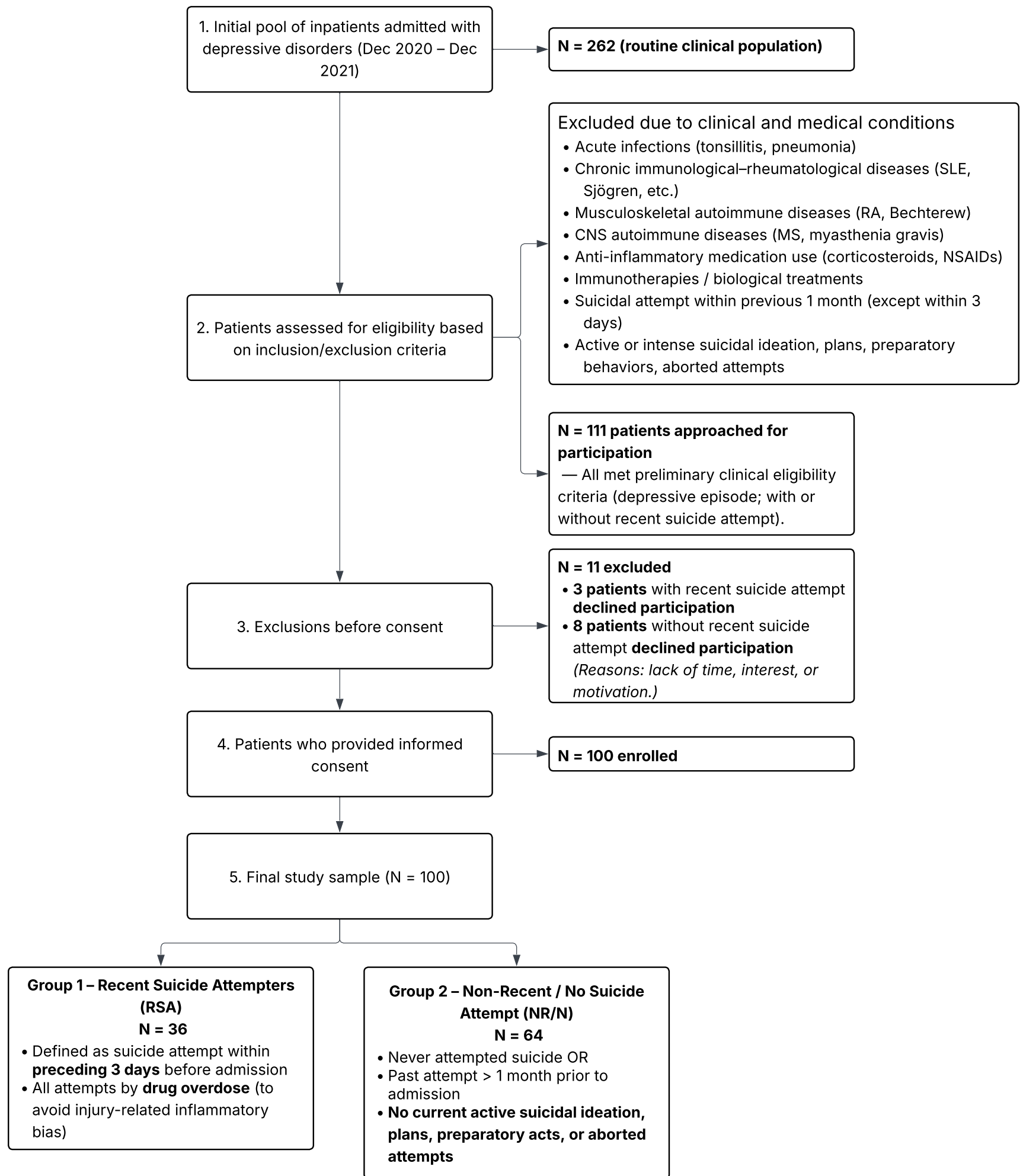

Supplement: Supplementary file 3 [file DataSheet2.pdf]
